# Identification and pathogenicity analysis of *Fusarium* spp. on peach in China

**DOI:** 10.1186/s12866-023-02958-y

**Published:** 2023-08-07

**Authors:** Jingping Dong, Hengsong Shi, Yu Wu, Lina Yang, Feng Zhu, Zhaolin Ji

**Affiliations:** College of Plant Protection, Yangzhou University, Yangzhou, 225009 China

**Keywords:** Peach branches, Vascular bundles, *Fusarium* spp., Comparison of pathogenicity

## Abstract

**Background:**

Vascular system is affected by diseases that can seriously damage plant health by inducing browning and death of branches. This study aimed to identify and analyze the pathogenicity of *Fusarium* spp. isolates obtained from diseased peach branches in several peach-producing areas of China.

**Results:**

We obtained and confirmed nine *Fusarium* isolates based on morphological and molecular characteristics. Phylogenetic relationships using a combination of rDNA-internal transcribed spacer (ITS), elongation factor (EF)-1*α*, and mitochondrial small subunit (mtSSU) gene sequences were analyzed. GJH-Z1, GJH-6, and GJH-1 were identified as *F. avenaceum*; HYR-Z3, and ZLZT-6 as *F. concentricum*, HH-2020-G2, and HYTZ-4 as *F. solani*, GG-2020-1 as *F. asiaticum*, SYGZ-1 as *F. equiseti*. Through acupuncture comparison, the pathogenicity of *F. equiseti* (SYGZ-1) was highest amongst nine strains. Meanwhile, *F. concentricum* (HYR-Z3 and ZLZT-6), and *F. solaini* (HYTZ-4) had a higher level of pathogenicity as revealed by impregnation.

**Conclusions:**

Our study shed light on the findings that *Fusarium* spp. can inflict vascular bundle browning of peach plants. Our results will extend the understanding of pathogenic diseases in China’s peach industry.

**Supplementary Information:**

The online version contains supplementary material available at 10.1186/s12866-023-02958-y.

## Introduction

Peach (*Prunus persica* L.), originated in China and has adapted to various climates worldwide [1]. The Food and Agricultural Organization estimates show that China has been the largest peach producer in the world, accounting for 61.12% of global peach production in 2020 (Food and Agricultural Organization of the United Nations, accessed on February 14, 2022). At present, the main diseases of peach include brown rot, anthracnose, scab, bacterial shot hole, gummosis, powdery mildew, branch blight, and so on. The pathogens causing these diseases which can reduce peach yield seriously have been studied extensively [2–4]. *Fusarium* spp. is a class of notorious pathogens, causing rotting of the roots, trunk, flowers, branches, and fruits. It mainly infects the vascular bundle, destroys tissues and produces toxins, causing the wilting and death of host plants [5, 6]. Recent studies reported that *Fusarium* spp. causing the fruit rot of peaches in China [7, 8], Korea [9], and Pakistan [10]. However, how *Fusarium* spp. inspire vascular bundle diseases in peach is missing.

The morphological and molecular analysis can be an effective way to identify *Fusarium* spp. At present, the most common sequences used to confirm *Fusarium* spp. are portions of sequences encoding internal transcribed spacer (ITS) [11] in the ribosomal repeat region, translation elongation factor-1*α* (EF-1α) [12], intergenic spacer region [13], *β*-tubulin [14], calmodulin [15], and mitochondrial small subunit (mtSSU). The EF-1*α* gene was more suitable than mtSSU to analyze the systematic information of *Fusarium* spp [14]. Also, the same study showed that the combination of EF-1*α* and mtSSU gene sequences could better resolve other locus sequences encoding ITS, *β*-tubulin, and calmodulin genes. Several recent studies focused on the diseases caused by *Fusarium* spp. on peach in different areas. Zhang et al. [7] isolated *F. equiseti* in peach fruit with fluffy white masses and brown necrotic lesions using ITS. *F. avenaceum* detected via ITS, EF-1*α*, and *β*-tubulin in Korea causing peach fruit rot by engulfing it white- and purple-colored mycelial mat [9]. *F. solani* was isolated and identified in peach fruit based on the morphological characteristics and molecular identification using ITS and EF-1*α* in Hunan Province, China [8]. *F. proliferatum* was identified and isolated in Fujian Province, China [16]. In Pakistan, Khan et al. [10] isolated *F. sporotrichioides* in peach fruit. Much attention has been paid to the harm caused by *Fusarium* spp. to peach fruit. *Fusarium* spp. can infect and spread in the vascular bundle, causing peach withering, significantly impacting the peach industry.

We collected diseased branches from several peach-producing areas in China and isolated a variety of *Fusarium* spp. to understand their infection pattern and mechanism. We determined the species status of these strains and the pathogenicity on peach branches. Also, we examined the potential infestation pattern of *Fusarium* spp. on peach branches.

## Materials and methods

### Sampling and isolation of *Fusarium* spp.

From March to August 2020, the death of branches and the discoloration of vascular bundles were reported in several peach-producing areas of China. The isolates recovered from the dead branches using potato dextrose agar (PDA) were single-spored and transferred to a new PDA.

### Morphological characterization of the fungal strains

A 4-mm puncher punched holes on *Fusarium* sp. plates cultured on a new PDA in an incubator at 28 °C. Colonies’ color and growth diameter were observed and recorded for 15 days. Mycelia were picked from the edge of colonies using a sterile needle and cultured in an incubator at 25 °C. Then, the spore morphology was observed after 7 days using a biomicroscope (Carl Zeiss, Germany).

### Molecular identification of *Fusarium* spp.

#### DNA extraction

We extracted genomic DNA using hexadecyltrimethyl ammonium bromide (CTAB). Initially, 200 mg of colonies were placed in a 1.5-mL sterile microtube and treated with 850 µL of Tris-CTAB (100 mL of Tris, 280 mL of 5 mol/L NaCl, 40 mL of 0.5 mol/L Ethylene Diamine Tetraacetic Acid (EDTA), and 20 g CTAB, pH 8.0) in a thermostatic water bath at 65 °C for 1 h. The same volume (850 µL) of chloroform was added to the microtube with shaking up and down after cooling to room temperature. The microtube was centrifuged (3500 rpm, 5 min) after leaving the suspension undisturbed for 10 min. Then, 400 µL of the supernatant was carefully transferred to a new 1.5 mL sterile microtube. The supernatant with an equal volume of isopropyl alcohol was left undisturbed in a refrigerator at − 30 °C for 3 h. Following centrifugation at 3500 rpm for 20 min, 750 µL of 75% ethyl alcohol was added to the suspension and incubated at 3500 rpm for 15 min. The pellet was dried by placing the microtube in the fume hood with the lids open for a while. Finally, the pellets were resuspended in 100 µL of sterile ddH_2_O, following which the concentration and quality of DNA samples were checked using an ultraviolet spectrophotometer (Thermo Fisher Scientific, USA) and 1% agarose gel electrophoresis.

### Polymerase chain reaction

Polymerase chain reaction (PCR) amplification of the rDNA-ITS gene fragment, mtSSU gene fragment, and EF-1*α* gene fragment was performed using a set of universal primers synthesized by Tsingke (Nanjing, China), to clarify the sequence diversity of *Fusarium* spp. The primers were as follows: ITS4 5′-TCCTCCGCTTATTGATATGC-3′ and ITS5 5′-GGAAGGTAAAAGTCAAGG-3′ [17], NMS 1 5′-CAGCAGTGAGGAATATTGGTCAATG-3′ andNMS 2 5′-GCGGATC ATCGAATTAAATAACAT-3′ [18], and EF-1 5′-ATGGGTAAGGAAGACAAGAC-3′ and EF-2 5′-GGAAGTACCAGTGATCATGTT-3′ [19].

For each DNA sample, the 25 µL PCR reaction mixture comprised 1 µL of DNA sample, 12.5 µL of 2× Taq Mix (Vazyme, China), 1 µL of primer F (50 ng/µL), 1 µL of primer R (50 ng/µL), and 10.5 µL of diethylpyrocarbonate (DEPC) water. The DNA samples were PCR amplified using an Eppendorf Mastercycler Pro (Eppendorf, Germany) with the following temperature program: (rDNA-ITS) 95 °C for 2 min, followed by 33 cycles of 95 °C for 30 s, 55 °C for 30 s, and 72 °C for 2 min; (EF-1α) 95 °C for 2 min, followed by 33 cycles of 95 °C for 30 s, 54 °C for 30 s, and 72 °C for 1 min; and (mtSSU) 95 °C for 2 min, followed by 33 cycles of 95 °C for 1 min, 55 °C for 1 min, and 72 °C for 1 min. The final step was an extension at 72 °C for 10 min and then cooling to 4 °C. Subsequently, 5 µL of the PCR product was obtained and detected using polyacrylamide gel electrophoresis.

### Sequencing and phylogenetic analysis

Tsingke (Nanjing, China) performed the bidirectional sequencing of PCR products. SeqMan software in DNASTAR software was used for bidirectional splicing and manual proofreading to obtain more reliable sequences. These sequences were compared using the GenBank Basic Local Alignment Search Tool (BLAST) of the National Center for Biotechnology Information (NCBI), and nine strains of *Fusarium* spp. were obtained.

The BLAST results of rDNA-ITS, EF-1*α*, and mtSSU gene sequences were used as the basis (Table 1). We established the phylogenetic tree by sequentially connecting DNA-ITS, EF-1*α*, and mtSSU gene sequences using the neighbor-joining (NJ) method of molecular evolutionary genetics analysis, version 6.0 (MEGA6) software. The branches with more than 50% confidence interval were selected, and the existing sequences of *Fusarium* spp. in the NCBI database were used to estimate similarity percentages. The gene sequences of nine strains were deposited in the GeneBank. Their accession numbers are listed in Supplementary Table 1. The accession numbers of reference sequences are listed in Supplementary Table 2.

**Table 1:** Results of molecular identification of *Fusarium* spp

Number	rDNA-ITS		EF-1α		mtSSU		Molecular identification by phylogenetic tree
	Specific Name	Similarity	Specific Name	Similarity	Specific Name	Similarity	
GJH-Z1	*F. avenaceum*	93.12%	*F. avenaceum*	98.18%	*F. avenaceum*	100%	*F. avenaceum*
GG-2020-1	* F. graminearum*	99.07%	*F. asiaticum*	99.08%	*F. asiaticum*	99.23%	*F. asiaticum*
HYR-Z3	*F. solani*	99.27%	*F. concentricum*	99.54%	*Citrullus lanatus*	77.45%	*F. concentricum*
GJH-6	* F. avenaceum*	95.05%	*F. fredkrugeri*	73.83%	*F. avenaceum*	100%	*F. avenaceum*
ZLZT-6	* F. solani*	99.44%	*F. concentricum*	100%	*Elizabethkingia meningoseptica*	96.63%	*F. concentricum*
SYGZ-1	* F. equiseti*	99.24%	*F. concentricum*	97%	*F. equiseti*	99.73%	*F. equiseti*
HH-2020-G2	*F. solani*	99.46%	*F. solani*	99.86%	*Elizabethkingia meningoseptica*	98.21%	*F. solani*
GJH-1	* F. avenaceum*	99.45%	*F. concentricum*	84.80%	*F. avenaceum*	100%	*F. avenaceum*
HYTZ-4	* F. solani*	99.64%	*F. solani*	99.86%	*F. solani*	99.68%	*F. solani*

### Pathogenicity of *Fusarium* spp. on peach branches

Peach lines, named “Chunmei” (provided by Ziqiao fruit and vegetable picking garden in Wangyun village, Chenji town, Yizheng City, Yangzhou, China), were used to identify the pathogenicity of *Fusarium* spp. in this study. Koch’s rules were confirmed by re-isolating the different strains in the inoculated peach branches and pathogenicity tests were performed on healthy branches.

### Inoculation by acupuncture

The peach branches were cut into small segments with similar lengths on a clean bench, disinfected with alcohol for 1 min, and then cleaned with sterile water for 1 min. A small wound was made in the middle of each peach branch with a sterilized needle with three biological replicates. A 4-mm puncher with hyphae was placed at the wound site of each branch, and the pure PDA puncher was used as the control. The bottom end of each peach branch was wrapped with sterilized wet cotton to ensure that the branches were vital. The treated peach branches were arranged in a sterile box covered with sterile wet gauze at the bottom and sealed with plastic wrap to ensure a sterile environment. The pathogenicity of *Fusarium* spp. on peach branches placed in an incubator at 28 °C was observed and recorded for 7 days.

### Inoculation by impregnation

Five 4-mm punchers with hyphae were transferred to 200 mL of potato dextrose broth (PDB) in a shaker at 28 °C and 200 rpm for 2 days. The pure punchers were used as the control. The peach branches were cut into several small segments with similar lengths on a clean bench, disinfected with alcohol for 1 min, and then cleaned with sterile water for 1 min. The bottom end of each branch was inserted into a conical flask with PDB-containing punchers. The branches in a sterilized box sealed with a plastic wrap were cultivated in an incubator at 28 °C for 7 days. Subsequently, the branches were cut lengthwise with a sterile scalpel to be observed and recorded.

## Results

### Morphological characterization of *Fusarium* sp. isolates

Three isolates were obtained from the peach branches grown at Yuncheng, Shanxi Province. The SYGZ-1 *Fusarium* sp. with a 35.00% isolation rate was recorded in March 2020. A total of five isolates were obtained from the peach branches of Qingzhen, Guizhou Province in April 2020, three (GJH-1, GJH-6, and GJH-Z1) of which were *Fusarium* spp. with an isolation rate of 28.00%, 24.00%, and 64.96%, respectively. In April 2020, three isolates were obtained from the peach root of Handan, Hebei Province, one (HH-2020-G2) of which was *Fusarium* sp. with an isolation rate of 44.00%. Similarly, in May 2020, three isolates were obtained from the peach branches of Guiyang, Guizhou Province, one (GG-2020-1) of which was *Fusarium* sp. with an isolation rate of 10.00%. In March 2020, six isolates were obtained from the peach branches of Lishui, Zhejiang Province. Amongst the six isolates, one (ZLZT-6) was identified as *Fusarium* sp. with an isolation rate of 6.67%. In August 2020, two (HYTZ-4, HYR-Z3) *Fusarium* spp. out of five isolates with a respective isolation rate of 12.00% and 20.00% were obtained from the peach root at Yanling, Hunan Province (Table 2).

**Table 2:** Number and source of tested strains

Number	Source	Isolation Time
GJH-Z1	Guiyang, Guizhou	2020.04.26
GG-2020-1	Guiyang, Guizhou	2020.05.25
GJH-6	Guiyang, Guizhou	2020.04.23
GJH-1	Guiyang, Guizhou	2020.04.22
HH-2020-G2	Handan, Hebei	2020.04.17
HYR-Z3	Yanling, Hunan	2020.08.09
HYTZ-4	Yanling, Hunan	2020.08.09
SYGZ-1	Yuncheng, Shanxi	2020.03.22
ZLZT-6	Lishui, Zhejiang	2020.06.23

**Fig. 1:**
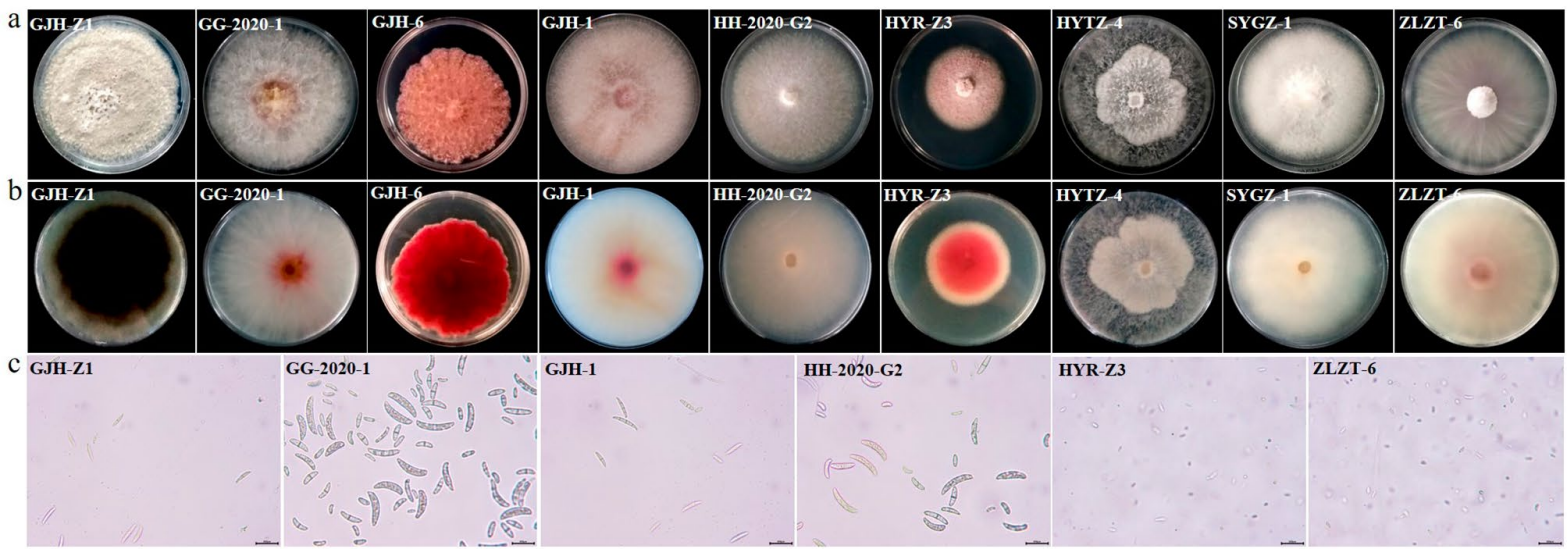
Colonial morphology of nine *Fusarium* spp. after 15 days of incubation on PDA at 28 °C. (**a**) Morphology of GJH-Z1, GG-2020-1, GJH-6, GJH-1, HH-2020-G2, HYR-Z3, HYTZ-4, SYGZ-1, and ZLZT-6. (**b**) Reverse morphology of the nine isolates, respectively. (**c**) Morphological appearance of the conidia of GJH-Z1, GG-2020-1, GJH-1, HH-2020-G2, HYR-Z3, and ZLZT-6.

**Fig. 2:**
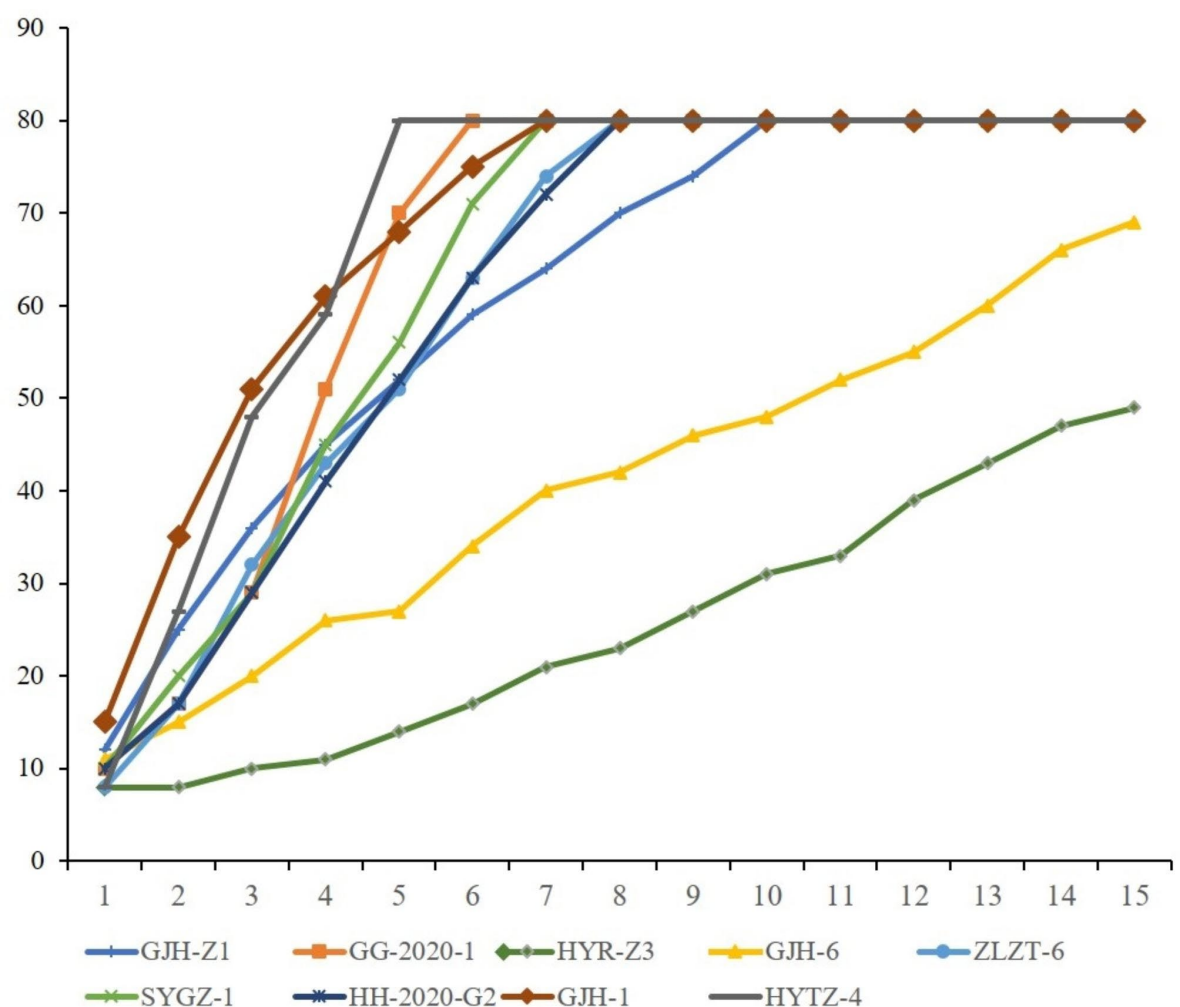
Colony growth of nine *Fusarium* spp

Nine strains of *Fusarium* spp. with browning capacity of vascular bundles were collected and isolated from several peach-producing areas in China. The colony morphology of the nine strains is depicted in Table 3. In terms of hyphae shape, GG-2020-1 were patchy, HYTZ-4 and ZLZT-6 were in mixed state, whereas other strains were villiform. The color of the colonies of GG-2020-1 and GJH-1 gradually varied. All strains were all whole-colored (Fig. 1a and b). The characterization process was repeated several times to avoid contamination. The individual growth rate of HYTZ-4 and GG-2020-1 was 18.00 mm/d and 13.80 mm/d. The growth rate of GJH-6 and HYR-Z3 was low, recording as 3.86 mm/d and 2.93 mm/d, respectively. Medium growth rate of ZLZT-6, SYGZ-1, HH-2020-G2, GJH-1, GJH-Z1 was 11.00 mm/d, 10.60 mm/d, 10.29 mm/d, 10.71 mm/d, 6.71 mm/d, respectively (Fig. 2). The mycelia of GG-2020-1 and HYTZ-4 grew all over an 80-mm-diameter petri dish for 5 days.

**Table 3:** Morphological identification of *Fusarium* sp. isolates obtained from different peach-producing areas

No. of Strains	Hypha	Colony color		Grown Rate	Macroconidium	
		Obverse	Reverse		Number of Septa	Size(µm)
GJH-Z1	Villiform	Gray	Black in center, gray in edge	M	4–5	80–340
GG-2020-1	Patcky	Yellow in center, white in edge	Red in center, white in edge	R	4–5	80–340
GJH-6	Villiform	Red	Red	S	-	
GJH-1	Villiform	Pink	Pink	R	4–5	80–340
HH-2020-G2	Villiform	White	Canary	M	4–5	80–340
HYR-Z3	Villiform	Pink	Red in center, pink in edge	S	0	10–45
HYTZ-4	Villiform in center, patcky in edge	White	White	R	-	
SYGZ-1	Villiform	White	Canary	M	-	
ZLZT-6	Villiform in center, filiform in edge	White	Canary	M	0	10–45

Note: M indicated middle; R indicated rapid; S indicated slow. “-” indicated that no conidia was found

We observed the morphology of spores using the biomicroscope to assist in identifying different strains of *Fusarium* spp. No spores were observed in GJH-6, SYGZ-1, and HYTZ-4. The spores of HYR-Z3 and ZLZT-6 were oval without septa, and the length of spores ranged from 10 to 45 μm. The remaining spores were all sickle-shaped with four to five obvious septa with the the length spores ranging from 80 to 340 μm (Fig. 1c).

### Molecular identification of *Fusarium* sp. isolates

We amplified with rDNA-ITS (ITS4 and ITS5), EF-1*α* (EF-1 and EF-2), and mtSSU (NMS1 and NMS2) universal primers to obtain the regional sequences of *Fusarium* spp. (Supplementary Fig. 1a–1c). PCR amplification using ITS4 and ITS5 provided fragments of 600 bp in size.The EF-1 and EF-2 with a fragments of 750 bp in size. Meanwhile, the NMS1 and NMS2 had different fragments as follows: the fragment of GJH-Z1, HYR-Z3, GJH-6, and SYGZ-1 was 750 bp in size, whereas GG-2020-1, ZLZT-6, and GJH-1 were 700 bp in size. The fragments of HH-2020-G2 were 650 bp in size, while the HYTZ-4 showed two bands of 750 and 650 bp in size. The DEPC water, as the negative control failed to show any band.

The rDNA-ITS, EF-1*α*, and mtSSU partial sequences retrieved from the NCBI database were analyzed to confirm the evolutionary relationship of *Fusarium* spp. The GenBank accession numbers of nine *Fusarium* strains are listed in Supplementary Table 1. From the BLAST search, sequences with high similarity were selected, and the species names were assigned according to the closest BLAST search (Table 1). For rDNA-ITS partial sequences, the species name of GJH-Z1, GJH-6, and GJH-1 was *F. avenaceum* with a similarity of 93.12%, 95.05%, and 99.45%, respectively. The HYR-Z3, ZLZT-6, HYTZ-4, and HH-2020-G2 was named as *F. solani* with a similarity of 99.27%, 99.44%, 99.64%, and 99.46%, respectively. The GG-2020-1 was enlisted as *F. graminearum* with a similarity of 99.07%. The SYGZ-1 was labeled as *F. equiseti* with a similarity of 99.24%. For EF-1*α* partial sequences, the ZLZT-6, HYR-Z3, SYGZ-1, and GJH-1 were tagged as *F. concentricum* with a similarity of 100%, 99.54%, 97.00%, and 84.80%, respectively. The HH-2020-G2 and HYTZ-4 were named as *F. solani*, with a similarity of 99.86%. With a similarity of 98.18%, the GJH-Z1 was listed as *F. avenaceum*. The GG-2020-1 and GJH-6 were respectively named as *F. asiaticum* and *F. fredkrugeri* with a similarity of 99.08% and 73.83%. For mtSSU partial sequences, the GJH-Z1, GJH-6, and GJH-1 were labeled as *F. avenaceum* with a similarity of 100%. With a similarity of 99.23%, the GG-2020-1 was named as *F. asiaticum*. The SYGZ-1 was named as *F. equiseti* with a similarity of 99.73%. The HYTZ-4 was titled as *F. solani* with a similarity of 99.68%. The HYR-Z3 with a similarity of 77.45% was named as *Citrullus lanatus*. The ZLZT-6 and HH-2020-G2 were respectively titled as *Elizabethkingia meningoseptica* with a similarity of 96.63% and 98.21%.

**Fig. 3:**
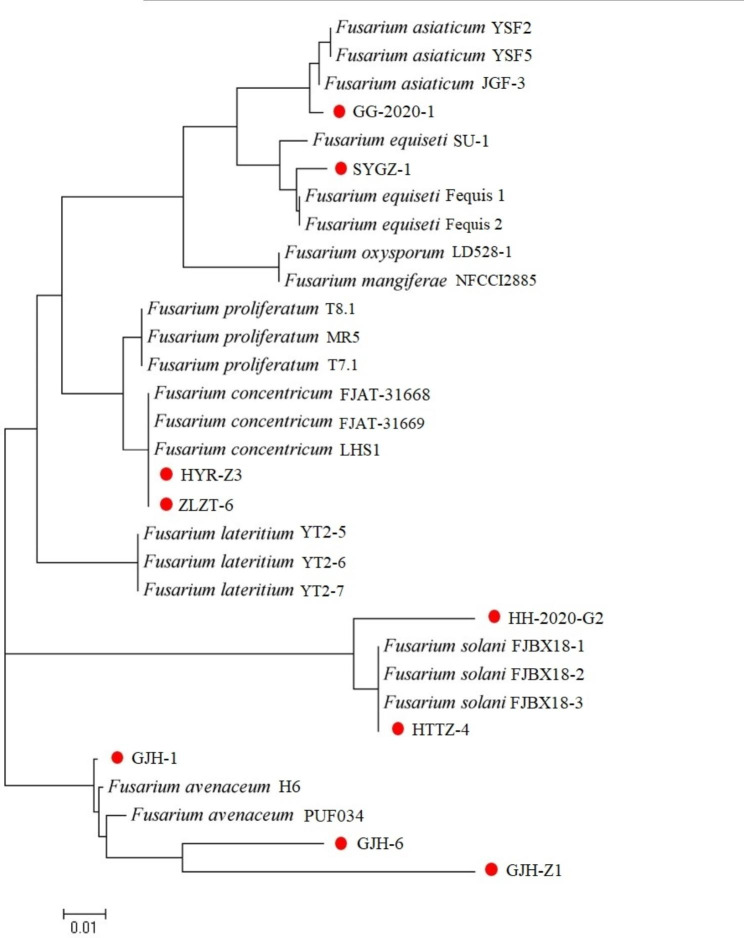
Phylogenetic tree of the combination of rDNA-ITS, EF-1*α*, and mtSSU genes of *Fusarium* sp. isolates by the NJ method using MEGA6.

A phylogenetic tree was constructed using the MEGA6 employing the NJ method to understand the evolutionary relationship of the *Fusarium* spp. (Fig. 3). The GenBank accession numbers of these referenced sequences of *Fusarium* spp. are provided in Supplementary Table 2. The tree indicated that GJH-1, GJH-Z1, and GJH-6 formed a monophyletic cluster with *F. avenaceum* H6 and *F. avenaceum* PUF034. We hypothesized that the GJH-1, GJH-Z1, and GJH-6 could be placed in *F. avenaceum* category. SYGZ-1 formed a monophyletic cluster with *F. equiseti* isolate SU-1, *F. equiseti* isolate Fequis1, and *F. equiseti* isolate Fequis 2. *F. equiseti* could be proposed as the species name of SYGZ-1. HH-2020-G2, and HYTZ-4 formed a monophyletic cluster with *F. solani* FJBX18-1, *F. solani* FJBX18-2, and *F. solani* FJBX18-3. The *F. solani* could be projected as the species name of HH-2020-G2, and HYTZ-4. ZLZT-6 and HYR-Z3 formed a monophyletic cluster with *F. concentricum* FJAT-31,668, *F. concentricum* FJAT-31,669, *F. concentricum* LHS1. The *F. concentricum* could be anticipated as the species name of ZLZT-6 and HYR-Z3. GG-2020-1 formed a monophyletic cluster with *F. asiaticum* YSF2, *F. asiaticum* YSF5, *F. asiaticum* JGF-3. *F. asiaticum* could be proposed as GG-2020-1.

### Pathogenicity of *Fusarium* spp. on peach branches

**Fig. 4:**
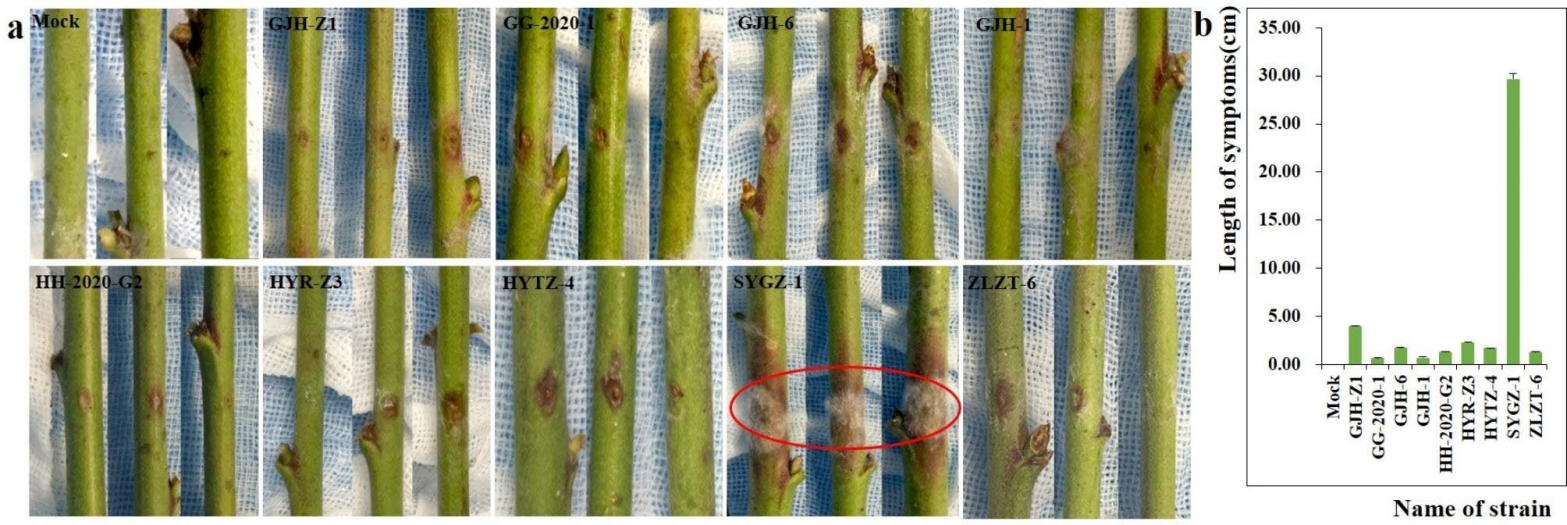
Symptoms of peach branches inoculated by the acupuncture method. (**a**) Epidermal symptoms of peach branches after inoculation. (**b**) Histogram of the length of epidermal lesions of peach branches

A single colony was selected and purified to check its pathogenicity on healthy peach branches. Four-millimeter punchers with hyphae were used for the acupuncture method. Nine isolates of *Fusarium* spp. could cause oval brown lesions on the wound of the peach branches with dense mycelium on the surface of lesions (Fig. 4A). Among these, the longitudinal diameter of the lesion (30 mm) caused by SYGZ-1 was much higher than that of the lesions caused by other isolates (all less than 5 mm) (Fig. 4B). The branches pressed with the control puncher had no symptoms. From the obtained results, we hypothesized that SYGZ-1 is the most pathogenic compared to others.

**Fig. 5:**
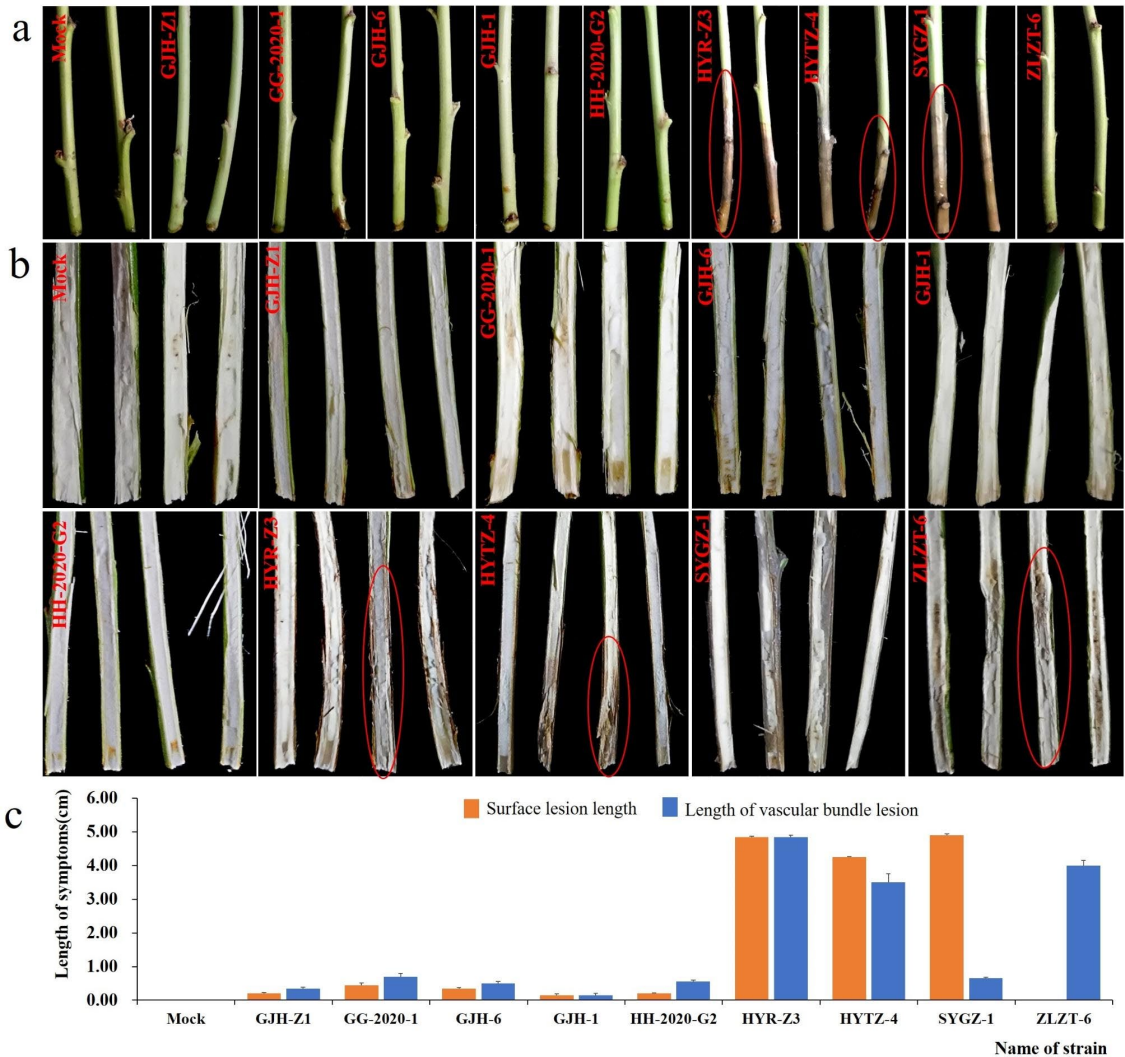
Symptoms of peach branches inoculated by the impregnation method. (**a**) Epidermal symptoms of peach branches after inoculation. (**b**) Symptoms of vascular bundles in the longitudinal section after inoculation. (**c**) Histogram of the length of epidermis and vascular bundle lesions on peach branches

The impregnation involved using a 4-mm puncher with hyphae transferred into PDB further and cultured in a shaker for 2 days. The surface of peach branches impregnated with ZLZT-6 was asymptomatic as the control. The branches impregnated with other isolates developed lesions of variable sizes (Fig. 5A C). Among these, HYR-Z3, SYGZ-1, and HYTZ-4 caused the largest lesions on peach branches. All nine isolates could infect and expand on the inner surface of peach branches, causing the browning of vascular bundles. HYR-Z3, ZLZT-6, and HYTZ-4 caused the largest number of lesions on the inner surface of branches (Fig. 5B C). We believed that HYR-Z3, SYGZ-1, and HYTZ-4 were the most pathogenic on the surface of peach branches compared with other isolates. However, HYR-Z3, ZLZT-6, and HYTZ-4 were the most pathogenic, as revealed by the lesion size and numbers.

## Discussion

In this study, nine *Fusarium* spp. isolates were obtained from peach branches showing symptoms such as browning of vascular bundles and branches withering. We also isolated a large number of other strains, as follows: *Nigrospora oryzae* in Lishui, Zhejiang Province; *Pestalotiopsis lushanensis* and *P. vismiae* in Qingzhen, Guizhou Province; *Pseudomonas baetica* and *Bacillus* sp. in Yuncheng, Shanxi Province; *Curtobacterium citreum*, *Rahnella aquatilis* and *Xylariales* sp. in Yanling, Hunan Province; and *Altemaria* sp. in Handan, Hebei Province. No studies have reported on the strains infecting peach at present, such as *N. oryzae*, *P. lushanensis*, *P. vismiae*, *P. baetica*, *C. citreum*, *R. aquatilis*, *Altemaria* sp., *Bacillus* sp. can reduced incidence and lesion diameter of peach brown rot caused by *Monilinia fructicola* [19]. Probably *Fusarium* spp. isolated from peach branches and roots were the major pathogens. However, the pathogenicity of other isolates on peach branches was not determined, indicating that these isolates might pose a potential risks. We also collected some browned branches (Supplementary Fig. 2) from other production areas, including as Beijing, Yunnan, Sichuan, and Jiangsu provinces, but failed to separate the effective strains.

Researchers [20] showed that combining regional sequences could better resolve individual gene sequences. We combined the rDNA-ITS, EF-1*α*, and mtSSU gene sequences for phylogenetic analysis. Nine strains of *Fusarium* spp. were isolated from the phylogenetic tree based on the combination of rDNA-ITS, EF-1*α*, and mtSSU gene sequences. For mtSSU partial sequences, HYR-Z3 was aligned as *C. lanatus*, and ZLZT-6 and HH-2020-G2 were associated *to E. meningoseptica*, a Gram-negative aerobic organism with yellow pigments that could infect humans [21].

The isolates were confirmed based on morphological characteristics and molecular identification. The morphological characteristics were used for molecular identification following rDNA-ITS, EF-1*α*, and mtSSU sequence analysis. As shown in Fig. 1, the macroconidia of GJH-Z1 and HH-2020-G2 showed a slightly elongated sickle shape compared with the stout large macroconidia of GG-2020-1 and HYTZ-4. The phylogenetic identification based on the combination of rDNA-ITS, EF-1*α*, and mtSSU gene sequences suggested that GJH-Z1, GJH-6 and GJH-1 were *F. avenaceum*, but only microconidia of the GJH-Z1 and GJH-1 were found. HYTZ-4 and HH-2020-G2 were orientated as *F. solani* reported on wheat in Iran [22], corn in Serbia [23], young peach trees with root rot and stem canker in Puerto Rico [24], peach fruit with rot in Hunan province of China [8], and peach fruit in Korea [9]. Additionally, *F. avenaceum* was reported on barley in Canada [25]. GJH-6 and SYGZ-1 with no conidia were confirmed as *F. avenaceum*, and *F. equiseti*, respectively. The *F. equiseti* was noted on rice in Argentina [26], barley in southern Europe [27], wheat in Iran [22], cumin in India [28], grafted watermelon in Korea [29], and peach fruit in Shanghai [7]. The *F. lateritium* was recorded on hazelnut fruit in Italy [30], *Morus alba* var. *pendula* in Hungary [31], and weed plants in Uzbekistan [32].

Some pathogenic strains such as *F. lateritium* also significantly influence gummosis disease in peach [21]. Some pathogenic strains such as *F. kyushuense*, *F. graminearum*, *F. proliferatum*, and *F. solani* can also cause peach fruit rot. In this study, SYGZ-1 identified as *F. equiseti* could cause gum flow after infecting peach branches. Accurate identification of *Fusarium* spp. isolated from infected branches might be significant for designing novel management strategies for peach pathogens.

## Conclusion

In this work, we isolated several *Fusarium* spp. from branches of many China’s peach-producing areas. Morphological and molecular characteristics identified nine strains of *Fusarium* spp. The GJH-Z1, GJH-6, and GJH-1 were identified as *Fusarium avenaceum*. The HYTZ-4, and HH-2020-G2 were recognized as *F. solani*. The SYGZ-1 was classified as *F. equiseti*. The HYR-Z3 and ZLZT-6 were associated to *F. concentricum*. The GG-2020-G2 was projected as *F. asiaticum*. Koch’s rules were confirmed by re-isolating the above strains. The pathogenicity of nine strains on peach branches was compared using the acupuncture method and impregnation method. The pathogenicity of SYGZ-1 was higher than other strains as revealed by the impregnation method. The HYR-Z3 and ZLZT-6, SYGZ-1, and HYTZ-4 had a higher level of pathogenicity according to the acupuncture method. This research helps to understand how *Fusarium* spp. infect peach vascular bundle which can affect the overall growth of peach trees. The identification and pathogenicity of *Fusarium* spp. will be the basis for improving prevention and control methods and timing.

### Electronic supplementary material

Below is the link to the electronic supplementary material.

**Supplementary Table 2** GenBank accession numbers of reference *F*. spp.

Supplementary Material 2

Supplementary Material 3

**Supplementary Fig. 1** Amplification products using the universal primers of rDNA-ITS (a), EF-1α(b), and mtSSU (c) genes. M indicates marker (5000 bp), C indicates control, and 1-9 indicate GJH-Z1, GG-2020-1, HYR-Z3, GJH-6, ZLZT-6, SYGZ-1, HH-2020-G2, GJH-1, and HYTZ-4, respectively

**Supplementary Fig. 2** Field symptoms of browned branches of peach. (a) Brown symptom at the base of branches. (b) Brown lesions around the bud and the dead of the extracted bud

**Supplementary Table 1** GenBank accession numbers of nine *Fusarium* spp. isolates

## Data Availability

All data and material are available upon request to correspondence author. All data has already been deposited in the National Center for Biotechnology Information (NCBI) database (www.ncbi.nlm.nih.gov/search/), and were assigned the accession numbers that list in Supplementary Tables 1, followed as: The ITS sequences’ accession number and EF1-α sequences’ accession number of GJH-Z1 were MT975267 and MW008032. The GG-2020-1’s were MT982617 and MW008033, respectively. The HYR-Z3’s were MT982620 and MW008034, respectively. The GJH-6’s were MT982621 and MW008035, respectively. The ZLZT-6’s were MT991106 and MW008039, respectively. The SYGZ-1’s were MT950125 and MW008036, respectively. The HH-2020-G2’s were MT982647 and MW008037,respectively. The GJH-1’s were MT982649 and MW008038, respectively. The HYTZ-4’s were MT991105 and MW008040, respectively.

## Abbreviations

ITS: rDNA-internal transcribed spacer,
EF-1 *α*: Elongation factor − 1*α*
mtSSU: Mitochondrial small subunit
PDA: Potato dextrose agar
CTAB: Hexadecyltrimethyl ammonium bromide
EDTA: Ethylene Diamine Tetraacetic Acid
PCR: Polymerase chain reaction
BLAST: Basic Local Alignment Search Tool
NCBI: National Center for Biotechnology Information
NJ: Neighbor-joining
PDB: Potato dextrose broth
