# Supplementary material for: Identification and pathogenicity analysis of Fusarium spp. on peach in China
Source: BMC Microbiol. 2023 Aug 7;23:211. doi: 10.1186/s12866-023-02958-y (PMC10405372; doi:10.1186/s12866-023-02958-y)
Supplement: Supplementary file 1 — Supplementary Table 2 GenBank accession numbers of reference F. spp. [file 12866_2023_2958_MOESM1_ESM.pdf]

**Supplementary Table 2** GenBank accession numbers of reference *F.* spp.

| Species name | GenBank accession | | |
|---|---|---|---|
| | ITS | EF1-α | mtSSU |
| *F. asiaticum* JGF-3 | MW423687 | MW685833 | MW423687 |
| *F. asiaticum* YSF2 | OM791679 | OL631287 | OM791679 |
| *F. asiaticum* YSF5 | MT416343 | MT419960 | MT416343 |
| *F. avenaceum* H6 | MT482504 | MT511741 | MT489689 |
| *F. avenaceum* PUF034 | HQ165937 | HQ165865 | HQ165829 |
| *F. concentricum* FJAT-31668 | .MH613763 | MH613766 | MH613763 |
| *F. concentricum* FJAT-31669 | MH613764 | MH613767 | MH613764 |
| *F. concentricum* LHS1 | MT9144U96 | MT920920 | MT914496 |
| *F. equiseti* Fequis1 | OP520923 | OP618093 | OP520925 |
| *F. equiseti* Fequis2 | OP520924 | OP618094 | OP520926 |
| *F. equiseti* SU-1 | MK680159 | MK692895 | MK738126 |
| *F. lateritium* YT2-5 | MF521449 | MF521452 | MF521455 |
| *F. lateritium* YT2-6 | MF521450 | MF521453 | MF521456 |
| *F. lateritium* YT2-7 | MF521451 | MF521454 | MF521457 |
| *F. mangiferae* NFCCI2885 | ON003498 | ON032393 | ON003498 |
| *F. oxyspor*um LD528-1 | MW418098 | MW423622 | MW418098 |
| *F. proliferatum* T7.1 | ON411518 | ON419860 | ON417433 |
| *F. proliferatum* T8.1 | ON411521 | KU508358 | ON417435 |
| *F. proliferatum* MR5 | MW600437 | MW670455 | MW670461 |
| *F. solani* FJBX18-1 | MN272356 | MN295047 | MN328054 |
| *F. solani* FJBX18-2 | MN272358 | MN295048 | MN328052 |
| *F. solani* FJBX18-3 | MN298761 | MN295049 | MN328053 |
