# Supplementary figures and images for: Identification and pathogenicity analysis of Fusarium spp. on peach in China
Source: BMC Microbiol. 2023 Aug 7;23:211. doi: 10.1186/s12866-023-02958-y (PMC10405372; doi:10.1186/s12866-023-02958-y)

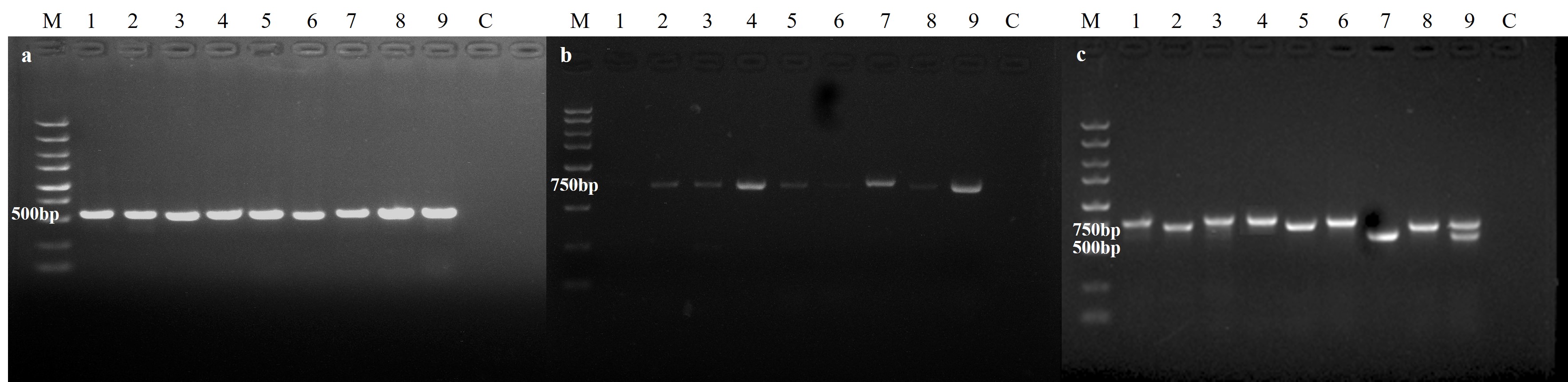

Supplement: Supplementary file 2 — Supplementary Material 2 [file 12866_2023_2958_MOESM2_ESM.jpg]

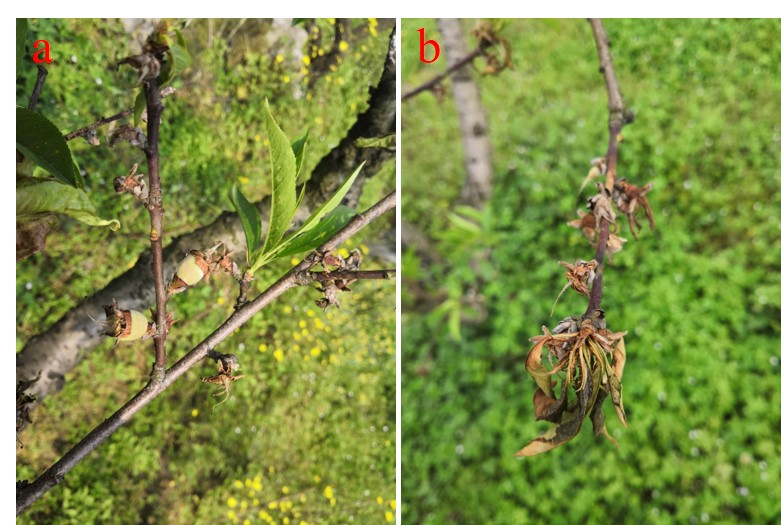

Supplement: Supplementary file 3 — Supplementary Material 3 [file 12866_2023_2958_MOESM3_ESM.jpg]
