# Supplementary material for: Identification and pathogenicity analysis of Fusarium spp. on peach in China
Source: BMC Microbiol. 2023 Aug 7;23:211. doi: 10.1186/s12866-023-02958-y (PMC10405372; doi:10.1186/s12866-023-02958-y)
Supplement: Supplementary file 4 — Supplementary Fig. 1 Amplification products using the universal primers of rDNA-ITS (a), EF-1α(b), and mtSSU (c) genes. M indicates marker (5000 bp), C indicates control, and 1-9 indicate GJH-Z1, GG-2020-1, HYR-Z3, GJH-6, ZLZT-6, SYGZ-1, HH-2020-G2, GJH-1, and HYTZ-4, respectively [file 12866_2023_2958_MOESM4_ESM.pdf]

Supplementary Fig. 1 Amplification products using the universal primers of rDNA-ITS (a), EF-1*a* (b), and mtSSU (c) genes. M indicates marker (5000 bp), C indicates control, and 1-9 indicate GJH-Z1, GG-2020-1, HYR-Z3, GJH-6, ZLZT-6, SYGZ-1, HH-2020-G2, GJH-1, and HYTZ-4, respectively.
