# Supplementary material for: Identification and pathogenicity analysis of Fusarium spp. on peach in China
Source: BMC Microbiol. 2023 Aug 7;23:211. doi: 10.1186/s12866-023-02958-y (PMC10405372; doi:10.1186/s12866-023-02958-y)
Supplement: Supplementary file 5 — Supplementary Fig. 2 Field symptoms of browned branches of peach. (a) Brown symptom at the base of branches. (b) Brown lesions around the bud and the dead of the extracted bud [file 12866_2023_2958_MOESM5_ESM.pdf]

Supplementary Fig. 2 Field symptoms of browned branches of peach. (a) Brown symptom at the base of branches. (b) Brown lesions around the bud and the dead of the extracted bud.
