# Supplementary material for: Identification and pathogenicity analysis of Fusarium spp. on peach in China
Source: BMC Microbiol. 2023 Aug 7;23:211. doi: 10.1186/s12866-023-02958-y (PMC10405372; doi:10.1186/s12866-023-02958-y)
Supplement: Supplementary file 6 — Supplementary Table 1 GenBank accession numbers of nine Fusarium spp. isolates [file 12866_2023_2958_MOESM6_ESM.pdf]

Supplementary Table 1 GenBank accession numbers of nine *Fusarium* spp. isolates

Species name	GenBank accession		
	ITS	EF1-α	mtSSU
F. avenaceum GJH-Z1	MT975267	MW008032	ns
F. asiaticum GG-2020-1	MT982617	MW008033	ns
F. concentricum HYR-Z3	MT982620	MW008034	ns
F. avenaceum GJH-6	MT982621	MW008035	ns
F. concentricum ZLZT-6	MT991106	MW008039	ns
F. equiseti SYGZ-1	MT950125	MW008036	ns
F. solani HH-2020-G2	MT982647	MW008037	ns
F. avenaceum GJH-1	MT982649	MW008038	ns
F. solani HYTZ-4	MT991105	MW008040	ns

Note: ns indicated that the accession numbers were not successfully obtained due to the few BLAST results for *Fusarium* using mtSSU sequences in GenBank.
